# Emergence of unusual human rotavirus strains in Salento, Italy, during 2006–2007

**DOI:** 10.1186/1471-2334-9-43

**Published:** 2009-04-15

**Authors:** Antonella De Donno, Tiziana Grassi, Francesco Bagordo, Adele Idolo, Alessandra Cavallaro, Giovanni Gabutti, the Collaborative Group for the surveillance of Rotavirus Infection

**Affiliations:** 1 Department of Biological and Environmental Sciences and Technologies, Lab of Hygiene, University of Salento, via prov.le Lecce-Monteroni, 73100 Lecce, Italy; 2 Department of Clinical and Experimental Medicine, Section of Hygiene and Occupational Health, University of Ferrara, Ferrara, Italy

## Abstract

**Background:**

In recent years, rotavirus genotyping by RT-PCR has provided valuable information about the diversity of rotaviruses (RV) circulating throughout the world.

The purpose of the present study was to monitor the prevalence of the different G and P genotypes of rotaviruses circulating in Salento and detect any uncommon or novel types.

**Methods:**

During the period from January 2006 to December 2007, a total of 243 rotavirus positive stool samples were collected from children with diarrhoea admitted to four Hospitals in the province of Lecce (Copertino, Galatina, Gallipoli and Tricase).

All the specimens were tested for RV by real time PCR and genotyped for VP7 (G-type) and VP4 (P-type) gene by reverse transcription (RT) and multiplex PCR using different type specific primers.

**Results:**

In course of this study we identified 4 common G&P combinations viz. G2P[8], G1P[8], G2P[4] and G9P[8] amongst 59.8% of the typeable rotavirus positives.

Rotavirus G2P[8] was recognized as the most widespread genotype during the sentinel-based survey in Salento.

The detection of other novel and unusual strains, such as G2P[10], G4P[10], G8P[4], G9P[11] and G10P[8] is noteworthy.

Furthermore, a significant number of mixed infections were observed during the survey period but G3P[8] rotaviruses were not detected.

**Conclusion:**

This study highlights the genetic diversity among rotaviruses isolated from children in Salento and the emergence of some novel strains. Therefore, it is highly essential to continuously monitor for these strains so as to assess the impact of vaccines on RV strains circulating in Salento and understand the effect of strain variation on efficacy of presently available vaccines.

## Background

Rotavirus (RV), the most common cause of gastroenteritis in infants and young children, presents a capsid formed by three concentric layers. The outer protein layer is composed of VP4 and VP7, the two major antigens of the virus, and the middle layer is composed of VP6 molecules arranged as trimers. The central core is composed mainly of VP2 and contains 11 segments of double-stranded RNA and enzyme complexes responsible for the processes of RNA transcription and replication [1].

As the two gene segments that encode the outer capsid proteins (VP4 and VP7) segregate independently, a typing system consisting of both G and P genotypes has been developed. To date, 15 G and 26 P genotypes have been described [2,3].

The segmented nature of the RV genome provides an opportunity for genetic reassortment, or plays an important role in rotavirus diversity through genetic shift as demonstrated by many authors [4,5].

Worldwide surveillance of RV strains has demonstrated G1 to G4 genotypes with P[8] and P[4] genotypes to be the most common circulating RV [5,6]. However, several recent international studies have reported some unusual types (G5, G8 and G12) and rare combinations of G and P types [6-10].

G12 strains, in particular, had been detected during the previous study in 2004 in Salento and nucleotide sequences were deposited in database with accession numbers EF536025 and EF536026 respectively [11].

Surveillance studies and documentation of rotavirus G and P genotypes is necessary for comprehensive evaluation of evolution of new strains and assessing the capability of vaccines to provide heterotypic protection.

The objective of this study was to monitor the prevalence of the different G and P types circulating in Salento and to detect uncommon and novel types.

This report highlights the results gathered during the ongoing sentinel-based network surveillance in hospitals of Salento having a paediatric ward, since 2004, to understand the molecular epidemiological changes amongst rotaviruses in relation to their G and P genotypes [12].

## Methods

From January 2006 to December 2007, a total of 243 rotavirus-positive stool specimens were collected during the course of treatment from children under 16 years of age hospitalized with gastro-enteric symptoms in four hospitals in the province of Lecce, specifically Copertino, Galatina, Gallipoli and Tricase.

The routine diagnosis for RV infection was carried out in the Clinical Virology Laboratories of the hospitals by means of rapid screening tests currently available in the market, such as latex agglutination (ROTAGEN, Biokit) and immuno-chromatographic (ROTA-STRIP QUICK-TEST, Amplimedical) tests. According to the producers, these tests have a sensitivity and specificity ranging between 95.7–100% and 96.3–100%, respectively. RV-positive specimens were collected anonymously in compliance with the Helsinki Declaration and with the Law Decree n. 196/2003, article 24 (Code for the protection of personal data). Furthermore, in this study was not performed any experimental work on patients, therefore the Ethics Committee of Ferrara (Italy) declared that this form of study doesn't need any consent by the Ethic Committee in compliance with the Italian Law.

The specimens were stored at -20°C until tested by molecular biology techniques.

The faecal samples (0.5–1 ml) were diluted to approx. 5 ml of 0.89% NaCl; centrifugated for 20 min at 3000 rpm and filtered using a 0.22 μm filter.

Rotavirus double-stranded RNA was extracted using the QIAamp Viral RNA kit (QIAGEN AG, Basel, Switzerland) according to the manufacturer's instructions.

The RNA was retro-transcribed and amplified by real-time PCR (Fastset Rotavirus; Arrows Diagnostics, Italy).

Reverse transcription with random primers and G and P typing semi-nested multiplex PCR were performed as described previously by Iturriza-Gomara et al [13].

The consensus primers (VP7-F/VP7-R and con2/con3) were used in multiplex first round reactions and type-specific primers (G1–G4, G8–G10 and P[4], P[6], P[8]–P[11]) were used in the second round reactions.

All the RT-PCRs were performed with viral RNA extracted from reference samples as positive controls and RNase free water as negative control.

RT-PCR fragments were electrophoresed in 2% agarose gels in Tris-borate-EDTA buffer along with a 100-bp or 50-bp DNA ladder (PROMEGA) as a standard marker. The gels were stained with ethidium bromide and amplicons were viewed with UV light.

## Results

A total of 243 stool samples were collected from children with acute gastroenteritis during 2006 (n = 108) and 2007 (n = 135).

All samples screened were found to be rotavirus positive by real time PCR. Among these 7 specimens were non typeable (i.e., neither a G nor a P genotype could be identified) by multiplex-PCR. Forty-eight samples were partially typed (i.e., only one of the two genotypic specificities was obtained; in 44 samples, no P type could be identified, and in 4 samples, no G type was identified). A total of 188 specimens were characterized according to G and P specificities (152 = G&P single typed and 36 = G&P mixed typed) (Table 1).

**Table 1:** The genotype nature of RV positive specimens

*Year*	*Rotavirus positive specimens minus untypeable*	Only G typed		Only P typed		G & P typed	
		
		*Single*	*Mixed*	*Single*	*Mixed*	*Single*	Mixed *
**2006**	108-3 = 105	17	4	3	-	59	22
**2007**	135-4 = 131	19	4	1	-	93	14
**2006–2007**	**243-7 = 236**	**36**	**8**	**4**	**-**	**152**	**36**

* Gmixed-P; G-Pmixed and Gmixed-Pmixed.

RV positive specimens which were G typed and/or P typed (single or mixed types) during 2006–2007

**Figure 1:**
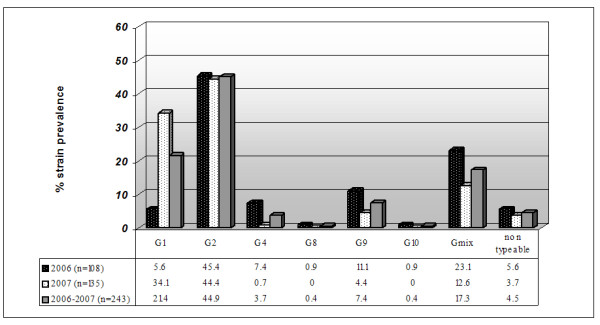
**Characterization of the G-types during 2006–2007**. Distribution of G genotypes (G1 to G4, G8 to G10, mixed [Gmix], and non typeable) among children hospitalized with acute rotavirus diarrhea in Salento.

RV genotypes G1 and G2, were observed to be widely circulating in Salento, with overall incidences of 21.4%, 44.9%, respectively. G4 (3.7%) and G9 (7.4%) strains were detected less frequently. Additionally, for the first time in Salento and only in 2006, G8 (0.4%, i.e. 1/243) and G10 (0.4%, i.e. 1/243) strains were detected. The G-type distribution in each year is described in figure 1.

**Figure 2:**
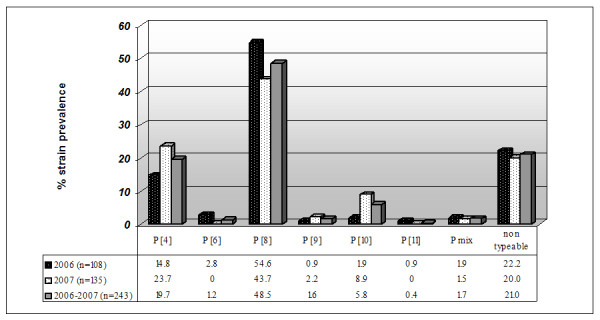
**Characterization of the P-types during 2006–2007**. Distribution of P genotypes (P[4], P[6], P[8] to P[11], mixed [Pmix], and non typeable) among children hospitalized with acute rotavirus diarrhea in Salento.

Among the P-types, P[4] and P[8] accounted for 19.7% and 48.5% of the total samples, respectively. Even in this case, we identified uncommon genotypes, such as P[9], P[10] and P[11] with overall incidences of 1.6% (4/243), 5.8% (14/243) and 0.4% (1/243), respectively. Instead, RV genotype P[6] was detected in three samples only in 2006 (Figure 2).

**Figure 3:**
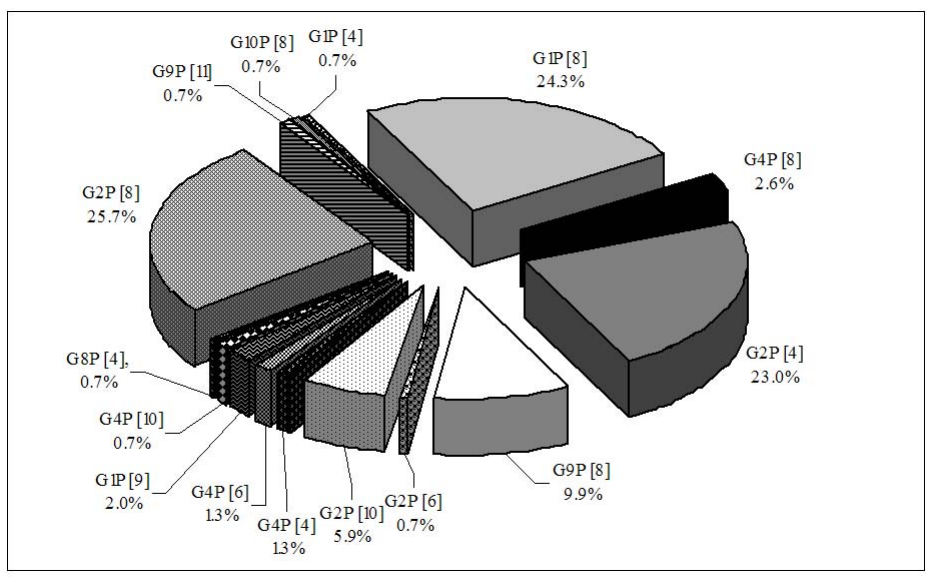
**Rotavirus G/P combinations circulating in Salento during 2006–2007**. Percentage of isolation of predominant rotavirus G/P combinations (single types) circulating in Salento during 2006–2007.

Totally, fifteen G/P combinations were identified, and the most common RV strains were G2P[8], G1P[8], G2P[4] and G9P[8] with prevalence of 25.7%, 24.3%, 23% and 9.9% of the typeable RV samples (n = 152), respectively. Less common types were G4P[8], G1P[9], G4P[6], G4P[4], G1P[4] and G2P[6], found in 2.6%, 2%, 1.3%, 1.3%, 0.7% and 0.7%, respectively. Besides, in this study novel and unusual RV strains, such as G2P[10] (5.9%), G4P[10] (0.7%), G8P[4] (0.7%), G9P[11] (0.7%) and G10P[8] (0.7%) were occasionally detected (Figure 3).

Furthermore, a low percentage of mixed infections was also present during the period 2006–2007, including 17.3% (42/243) among G types and 1.7% (4/243) for P types (Table 2).

**Table 2:** Distribution of RV mixed infections during 2006–2007

	Strains with given genotype		
Genotype	2006	2007	2006–2007
	
	n. (%)	n. (%)	n. (%)
***G mixed infections***			
G1 G2	3 (12%)	7 (41.2%)	10 (23.8%)
G1 G4	3 (12%)	-	3 (7.1%)
G1 G9	1 (4%)	3 (17.6%)	4 (9.5%)
G2 G4	2 (8%)	1 (6%)	3 (7.1%)
G2 G8	4 (16%)	3 (17.6%)	7 (16.7%)
G2 G9	6 (24%)	3 (17.6%)	9 (21.4%)
G2 G10	1 (4%)	-	1 (2.4%)
G9 G10	1 (4%)	-	1 (2.4%)
G1 G2 G8	1 (4%)	-	1 (2.4%)
G1 G2 G9	1 (4%)	-	1 (2.4%)
G2 G4 G10	1 (4%)	-	1 (2.4%)
G2 G9 G10	1 (4%)	-	1 (2.4%)
**TOTAL**	**25**	**17**	**42**
			
***P mixed infections***			
P[4] P[8]	1 (50%)	2 (100%)	3 (75%)
P[6] P[8]	1 (50%)		1 (25%)
**TOTAL**	**2**	**2**	**4**

During 2006 there was a greater variability in the G-types than 2007.

## Discussion

The genetic diversity of RV strains circulating in Europe is also associated with emerging strains that vary between regions from year to year [9,14].

Molecular analysis of the VP7 gene revealed the prevalence of six different G-genotypes in Salento.

In particular, study data from this 2-year investigation indicates that the G2 rotavirus continue to be the most prevalent genotype as it was in the previous surveillance study conducted between 2004–2005 in Salento [11].

Moreover, our study confirmed the persistence of rotaviruses G9, identified in this area since 2004 [11]. Genotype G9, previously infrequently reported, has become quite common all over the world [5]. An increased prevalence of the G9 genotype has also been noted in Italy [15,16]. This result reinforces the possibility that G9 may represent the fifth globally important genotype to be considered in vaccination programs.

Furthermore, this study documents the first detection of G8 and G10 strains in Salento. These strains (normally associated with animals) have been sporadically recovered from humans in various geographical areas. Genotype G8 strains were first reported from young children with gastroenteritis in Indonesia [17]. Later these rotaviruses were also reported from Finland, Italy, Nigeria, Brazil [18-21] and other parts of the African continent, especially Malawi [22,23].

RV G10 genotype was first isolated in the UK in 1992 but recent epidemiological surveys have reported the presence of this strain in India [24].

With respect to VP4 gene, six different P-types were identified; P[8] was the prevalent type while P[9], P[10] and P[11] were detected for the first time in Salento.

At present, worldwide, five common G/P combinations of RV predominate, namely G1P[8], G2P[4], G3P[8], G4P[8] and G9P[8] [5, 9, 14]. Global reviews of the main G and P type combinations encountered in human infections have identified G1P[8] as the most prevalent type [5].

In this research, only 4 of the most common G/P combinations were identified (no G3P[8] was found), representing 59.8% of the typeable RV samples. However, G2P[8] was the most prevalent combination during 2006–2007, while G1P[8] was predominant one in 2007.

G2P[8] is a new and unusual G/P combination that in recent years (with G1P[4] and G4P[4]) has been detected at relatively high frequency from different parts of the world [5].

The detection of uncommon strains, such as G2P[10], G4P[10], G8P[4], G9P[11] and G10P[8], is significant.

Finally, according to multiplex PCR results, a lot of mixed infections, especially in the G-types, were identified. These mixed infections most likely represent naturally occurring reassortment among RV strains [4,5,25]. Anyway, the use of multiplex PCR in the identification of mixed infectious should be handled with caution. The possibility of unspecific primer binding has to be taken into consideration. Furthermore, in mixed infections the rotavirus strains might be present at different concentrations, resulting in an uneven degree of PCR amplification, making the interpretation of the gel band pattern difficult [26,27].

In light of these considerations, additional experiments are necessary to confirm the results obtained during G and P typing. In particular, the confirmation of the high frequency of unusual G/P combinations found by sequence analysis is recommended.

## Conclusion

The diversity of strains circulating in different regions may be important for vaccine administration and development. Two live oral vaccines have recently been licensed to protect children against RV diarrhea: Rotateq, a human-bovine pentavalent vaccine, and Rotarix, a G1P[8] monovalent human rotavirus vaccine [28,29].

Clinical trials of these vaccines among children of rich and middle-income families in different countries have demonstrated high efficacy against the most common RV strains.

Anyway, the importance of heterotypic protection has to be fully understood; for this reason, virological surveillance and strain characterization should be implemented.

Post-marketing surveillance studies are needed to monitor the vaccine impact on circulating strains in order to evaluate the immunological pressure obtained by vaccination, to identify if strain replacement occurs, and to measure the extent of cross-protection against different RV genotypes, including G9 (which is becoming increasingly important in the world) and G8 (whose prevalence is increasing in different parts of Africa), as these G types were also detected in Salento.

## Competing interests

The authors declare that they have no competing interests.

## Authors' contributions

ADD, TG and GG conceived of the study and participated in its design and coordination and helped to draft the manuscript. TG and AI carried out the molecular studies. TG, FB and AC acquired and analyzed data and have been involved in revising the manuscript critically for intellectual content. All authors read and approved the final manuscript.

## Pre-publication history

The pre-publication history for this paper can be accessed here:

http://www.biomedcentral.com/1471-2334/9/43/prepub
